# Colonization, mortality, and host cytokines response to enterohemorrhagic *Escherichia coli* in rabbits

**DOI:** 10.18632/oncotarget.20966

**Published:** 2017-09-16

**Authors:** Mengjiao Guo, Wenhao Yang, Fahao Wu, Guangen Hao, Rong Li, Xinyu Wang, Liangmeng Wei, Tongjie Chai

**Affiliations:** ^1^ College of Animal Science and Veterinary Medicine, Sino-German Cooperative Research Centre for Zoonosis of Animal Origin of Shandong Province, Shandong Provincial Key Laboratory of Animal Biotechnology and Disease Control and Prevention, Shandong Provincial Engineering Technology Research Center of Animal Disease Control and Prevention, Shandong Agricultural University, Tai’an, Shandong, China; ^2^ Collaborative Innovation Center for the Origin and Control of Emerging Infectious Diseases, Taishan Medical University, Taian City, China

**Keywords:** enterohemorrhagic Escherichia coli, pathogenicity, innate immunity, cytokines, rabbits, Immunology and Microbiology Section, Immune response, Immunity

## Abstract

The major virulence factor of enterohemorrhagic *Escherichia coli* in infections is its ability to cause attaching and effacing lesions in enterocytes, as well as to produce Shiga toxins. To clarify the pathogenic mechanism and host innate immune responses of enterohemorrhagic *Escherichia coli* in rabbits, experimental infections with TS and MY strains were conducted. Among the results, although the MY strain’s pathogenicity was stronger than the TS, typical symptoms were observed in both groups of bacterial-infected rabbits. Pathological changes in the heart, liver, and spleen of rabbits infected with the MY strain were more severe than those infected with the TS strain, pro-inflammatory cytokines IL-1β, IL-6, IL-8, IFN-γ, and TNF-α were induced by both strains, and α- and β-defensin were significantly upregulated at 3 d postinfection. Moreover, in the spleen, the MY strain induced greater expressions of α- and β-defensins than did the TS strain. However, in the liver, the TS strain induced greater expressions of α- and β-defensins than did the MY strain. Most likely, different replications of the MY and TS strains in the liver and spleen induced different host immune responses. Altogether, the findings provide new insights into the occurrence and development of enterohemorrhagic *Escherichia coli*-mediated diseases in rabbits.

## INTRODUCTION

Enterohemorrhagic *Escherichia coli* (EHEC) belongs to the family of Shiga toxins (Stxs)-producing *E. coli*, many of whose members carry the eae gene and can cause severe disease [1]. As A-B-type toxins, Stxs are considered to be major virulence factors in the pathogenesis of haemolytic uremic syndrome and hemorrhagic colitis [2]. Similar to enteropathogenic *E. coli* (EPEC), EHEC causes attaching and effacing lesions, which represent another major virulence factor in colonization of the EHEC in the colon [3]. Enterohemorrhagic *Escherichia coli* (EHEC) was first isolated in 1977 by Konowalchuk et al [4]. In 1982, the bacterium first caused an outbreak of hemorrhagic enteritis in North America and then was named EHEC [5]. In Japan, the EHEC infection broke out in 1996. More than 9000 people were infected, resulting in 11 deaths. The event has attracted worldwide attention [6]. In United States, an outbreak of EHEC infection occurred in 2006. It was found that the EHEC contaminated spinach. 117 people were eventually infected and 3 died [7]. The outbreak of EHEC in developed countries such as the United States, Japan, Canada and the United Kingdom showed a trend of increasing year by year.

The most significant sources of EHEC infection in humans are food and water [8-10], the primary source of EHEC infection is the cattle in ruminants [11]. With the increased use of rabbits as a food source and in research, as well as their increased activity in large wildlife populations, the risk and likelihood of interspecies pathogen transmissions have also risen [12]. For one, EHEC transmission can occur between cattle and wild rabbits [13, 14]. Rabbits are a new reservoir host of EHEC that may pose a zoonotic risk for humans [15]. In response to the heightened risk, several animal models of EHEC-induced disease have been developed. When the newly emergent pathogenic *E. coli* was first studied more than 30 years ago, experiments involved EHEC O157:H7 using piglets [16, 17], and the oral administration of EHEC caused diarrhea as well as attaching and effacing lesions in the ileum, cecum, and colon of rabbits [18]. In the use of rabbits infected with rabbit-origin *E. coli* as experimental models of human disease, rabbits orally inoculated with EPEC strain RDEC-1 emerged as a good model for human EPEC infection [19], which simulates the development of infectious gastroenteritis in children [20]. Other studies have shown that natural and experimental infection with EHEC strains caused hemorrhagic diarrhea, cecal colitis, nephropathy, and hemolytic uremic syndrome in Dutch belted rabbits [1, 15, 21].

Stxs can induce classical mitogen-activated protein kinases and IL-8 when co-administered with TNF-α [22] and flagellin [23]. During the innate immune response process, pro-inflammatory cytokines induce immune cells differentiated from white blood cells that migrate to inflammatory sites, thereby eliminating pathogenic microorganisms. In turn, immune cells secrete pro-inflammatory cytokines such as IL-1β, IL-2, IL-6, IL-8, IFN-γ, and TNF-α. Different types of white blood cells, including natural killer cells and mononuclear macrophages, secrete cytokines and defensins *via* MyD88- and NF-κB-mediated signal pathways [24, 25] that promote lymphocyte proliferation and differentiation, as well as participate in the development of inflammation.

Antimicrobial peptides not only directly inhibit various microorganisms, but also can recruit immature dendritic cells and macrophages to reach the skin and mucosal tissues of microbial invasion through chemokine receptors, thus inducing specific immunity of pathogenic bacteria. Since their production is more than 100 times faster than that of IgM, they are also the first line of host defense [26]. Members of a large, diverse family of cationic antimicrobial peptides, defensins are strongly induced by inflammation or tissue damage. For example, the expression of human β-defensin-2 is rapidly induced in response to infection by intestinal pathogenic bacteria, and human β-defensin-3 is strongly upregulated in inflammatory diseases such as Crohn’s disease [27, 28]. Along with the direct killing of microorganisms, antimicrobial peptides can recruit and promote other elements of host immunity, particularly innate immunity [29, 30]. In other research, drosophila has responded to infection by synthesizing antimicrobial peptides (e.g., defensins) that, secreted into the hemolymph, exhibit antibacterial activity [24].

Although diseases in rabbits caused by EHEC have been reported [1, 15], research on the pathogenicity of EHEC and the role of innate immunity in disease processes remains scarce. What is the innate immune mechanism of rabbit resistance to EHEC infection? To investigate the inflammatory and anti-bacterial response, our research detected the major inflammatory cytokines and defensins. The purpose of this study is to systematically describe clinical symptoms and the histopathological analysis of *E. coli* content in tissues and innate immune responses in EHEC-infected rabbits.

## RESULTS

### Isolation of EHEC

**Figure 1:**
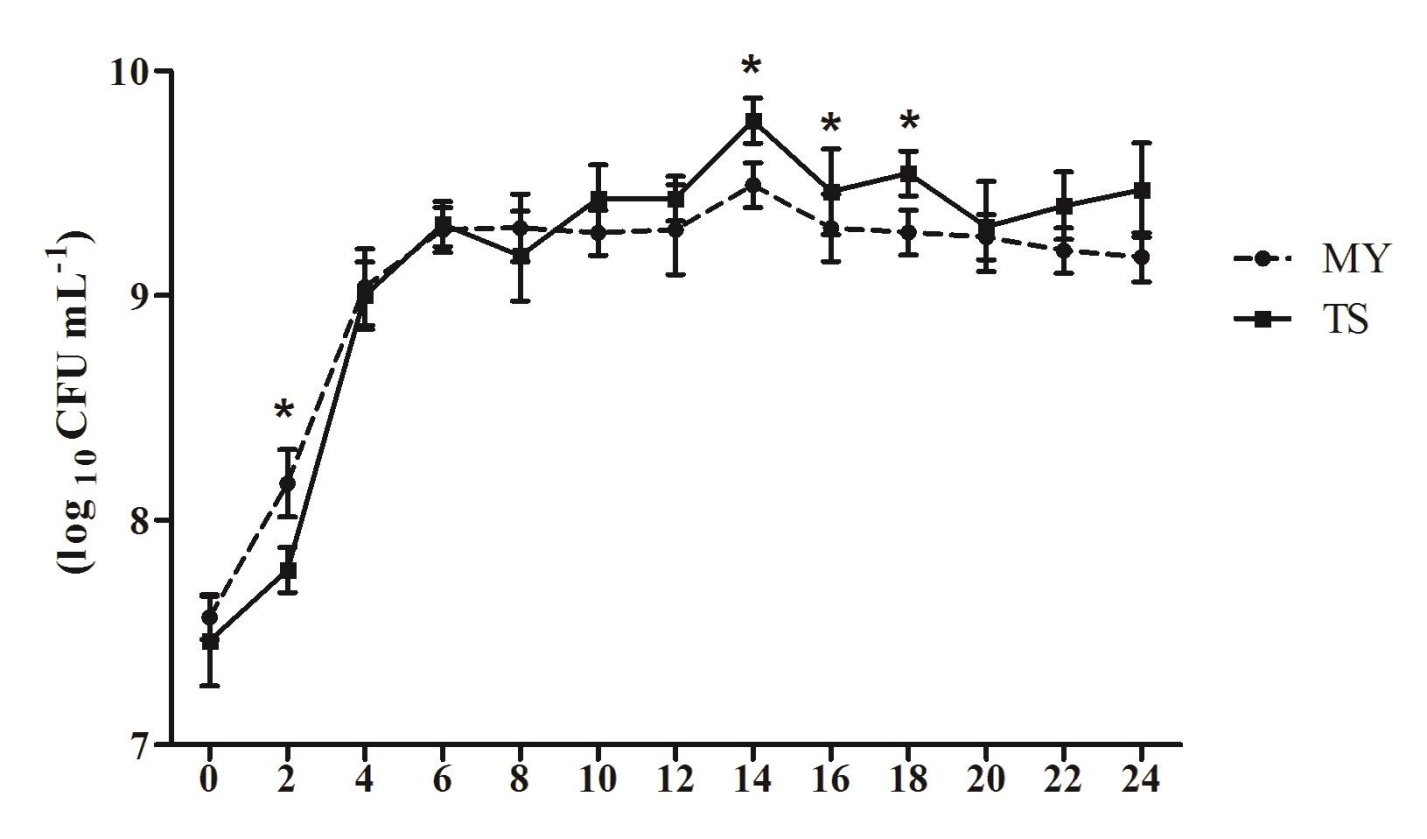
Growth curve of *E. coli* TS and MY strains After inoculation bacteria with 1% (v/v) seed liquid of the *E. coli* TS or MY, *E. coli* content was calculated every 2h. The data shows means ± SDs of three independent experiments. Significant differences are indicated with an *.

The bacterial colony of *E. coli* was bluish green with a metallic luster on the eosin-methylene blue medium. As shown in Table 2, the eae, Stx1, Stx2, and hlyA genes were all amplified from the MY strain; however, in the TS strain, the Stx2 gene was absent (Table 2). In addition, after 4 h of incubation, the number of *E. coli* MY rapidly reached 1.46 × 10^8^ CFU/mL, while that of TS was 5.99 × 10^7^ CFU/mL (*p* < 0.05). After 6 h of incubation, the number of *E. coli* MY and TS reached 1.96 × 10^9^ CFU/mL and 2.08 × 10^9^ CFU/mL, respectively. During the stable phase of bacterial growth, the highest number of *E. coli* TS reached 6.46 × 10^9^ CFU/mL, which was higher than that of MY 2.29 × 10^9^ CFU/mL at 14 h (*p* < 0.05). MY strain grew at a faster rate than the TS stain during the logarithmic growth phase, although its greatest concentration was lower than that of the TS stain (Figure 1).

### Survival rate and *E. coli* content

**Figure 2:**
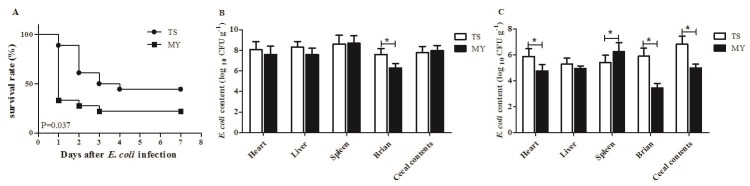
Rabbits were inoculated intraperitoneally with 1.0 mL of *E. coli* TS and MY bacterial suspension (10^8^ CFU/mL) **A.** The survival rate of rabbits after infection with the TS and MY strains (*n* = 10); **B.** *E. coli* content of infected rabbits at 1 dpi (log_10_ CFU/g); and **C.** *E. coli* content of infected rabbits at 3 dpi (log_10_ CFU/g). Bars represented the means ± SDs of three independent experiments (five rabbits per experiment). Significant differences are indicated with an *.

The incidence of diarrhea in rabbits infected with the MY strain was 80%, while that of rabbits infected with TS strain was 66.7%. When rabbits were infected with the MY strain, the number of deaths peaked at 1 d postinfection (dpi) dropped at 2 and 3 dpi, after which none of the rabbits died. By contrast, among rabbits infected with TS strain, the number of deaths peaked at 2 dpi (27.8%), and none of the rabbits died after 4 dpi. Altogether, the mortality of rabbits infected with the MY strain was 77.8%, which was greater than that of rabbits infected with TS strain (55.6%) (*p* < 0.05, Figure 2A).

*E. coli* content in the heart, liver, spleen, brain, and cecal contents was tested at 1 and 3 dpi. At 1 dpi, *E. coli* rapidly replicated in the tested tissues with elevated content, and *E. coli* content in the heart, liver, and brain infected with the TS strain were greater than those infected with the MY strain (Figure 2B). Especially, *E. coli* content in brain infected with the TS strain was 4.09 × 10^7^ CFU/mL, significantly higher than that of MY strain (2.03 × 10^6^ CFU/mL) at 1 dpi (*p* < 0.05, Figure 2B). At 3 dpi, the amount of *E. coli* in tested tissues had declined since 1 dpi. *E. coli* content in heart, brain, and cecal contents infected with the TS strain was greater than those infected with the MY strain at 3 dpi (*p* < 0.05, Figure 2C). However, after MY strain infection, the number of bacteria in the spleen was 1.89 × 10^6^ CFU/mL, higher than that of infected TS strain (2.60 × 10^5^ CFU/mL) at 3 dpi (*p* < 0.05, Figure 2C). Taken together, both *E. coli* could cause systemic infection in rabbits and spread to multiple organs, although the MY strain can do so more efficiently than the TS strain.

### Clinical symptoms and gross lesions

**Figure 3:**
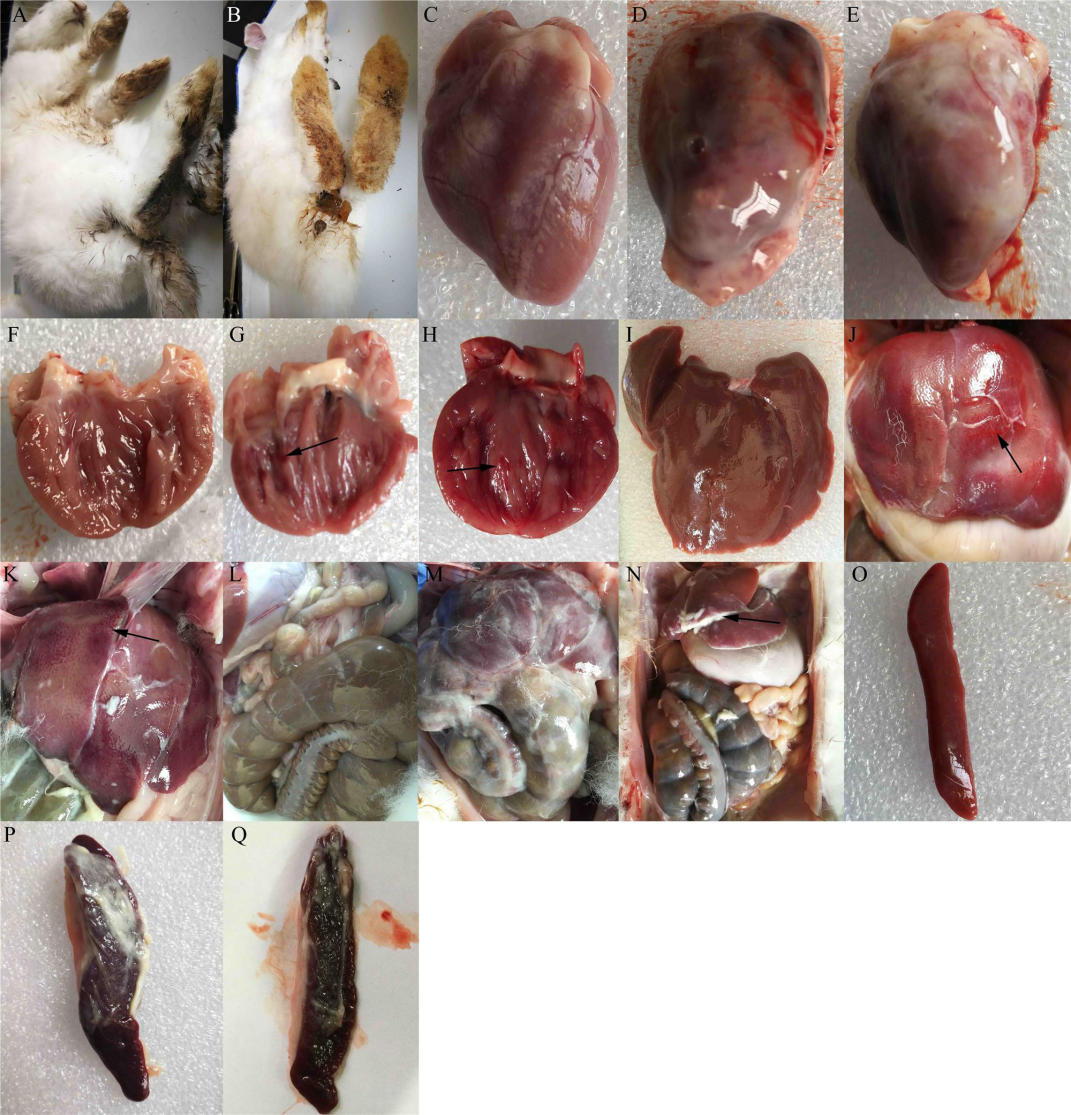
Clinical symptoms and gross lesions of rabbits after infection with enterohemorrhagic *Escherichia coli* Rabbits infected with the TS strain showed **A.** diarrhea, **D.** fibrinous pericarditis, **G.** endocardial hemorrhage, **J.** spotted hemorrhage of liver, **M.** abdominal cavity surface with white cellulose attachment, and **P.** spleen with cellulose attachment, splenomegaly, and hemorrhage. Rabbits infected with the MY strain showed **B.** diarrhea, **E.** fibrinous pericarditis, **H.** endocardial hemorrhage, **F.** spotted hemorrhage of the liver, **N.** a slight fibrinous exudate in the abdominal cavity, and **Q.** spleen with cellulose attachment, splenomegaly, and hemorrhage. Images C, F, I, L, and O show the epicardium, endocardium, liver, abdominal cavity, and spleen of the control group, respectively.

At 1 dpi, rabbits infected with either the MY or TS strain showed typical clinical symptoms, including mental depression, anorexia, and inactivity, as well as diarrhea (Figure 3A and 3B). At 3 dpi, they also showed typical gross lesions, including fibrinous pericarditis (Figure 3D and 3E), endocardial hemorrhage (arrow in Figure 3G and 3H), and liver and abdominal cavity surfaces with white cellulose attachments (Figure 3M and 3N). The Livers exhibited hemorrhage (arrow in Figure 3J and 3K), and the spleens were enlarged with fibrinous exudate and severe hemorrhage (Figure 3P and 3Q). No clinical symptoms were observed in the control group (Figure 3C, 3F, 3I, 3L, and 3O).

### Histopathological analysis

**Figure 4:**
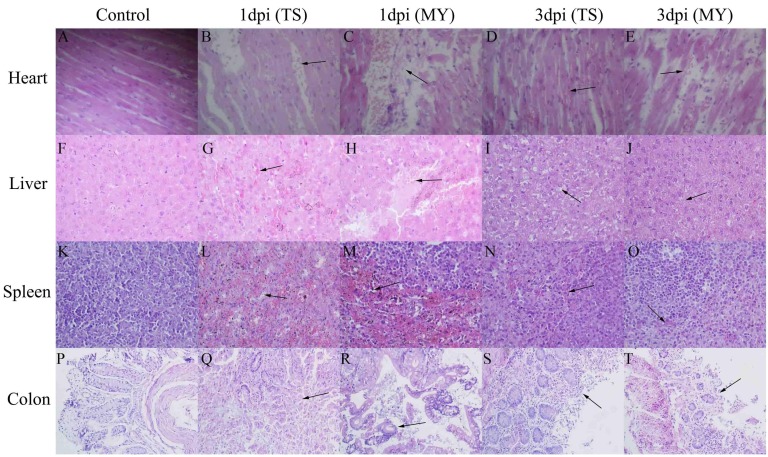
Pathological changes of the enterohemorrhagic *Escherichia coli*-infected rabbits at 1 and 3 d postinfection Image **B.** shows slight myocardial hemorrhage at 1 dpi, **C.** myocardial necrosis and hemorrhage at 1 dpi, **D.** myocardium hemorrhage at 3 dpi, **E.** hemorrhage, rupture, and necrosis of the myocardium at 3 dpi, **G.** hepatocyte hemorrhage at 1 dpi, **H.** hepatocyte necrosis with hemorrhage at 1 dpi, **I.** vacuolar degeneration, necrosis, and slight hemorrhage of hepatocytes at 3 dpi, **J.** vacuolar degeneration, necrosis, and hemorrhage of hepatocytes at 3 dpi, **L.** massive lymphocyte loss with diffuse hemorrhage at 1 dpi, **M.** massive lymphocyte loss with severe hemorrhage at 1 dpi, **N.** lymphocyte disappearance with hemorrhage at 3 dpi, **O.** a slight reduction of lymphocytes and hemorrhage at 3 dpi, **Q.** intestinal villus structure destruction at 1 dpi, **R.** intestinal villus fracture and goblet cell enlargement at 1 dpi, **S.** intestinal villus shedding at 3 dpi, and **T.** intestinal villus shedding at 3 dpi. Images A, F, K, and P show the heart, liver, spleen, and colon of rabbits from the control group, respectively; magnification of the heart, liver, and spleen tissues was 400×, whereas colon tissue was magnified 200×.

At 1 and 3 dpi, pathological changes occurred in various tissues in infected rabbits. Lesions in the heart, liver, and colon increased with time. Pathological changes in rabbits infected with the MY strain were more serious than those infected with the TS strain. Slight myocardial hemorrhage appeared in rabbits infected with the TS strain at 1 dpi (Figure 4B), and had grown severe by 3 dpi (Figure 4D). After being infected with the MY strain, rabbits clearly showed myocardial necrosis, rupture, and hemorrhage (Figure 4C and 4E). Hepatocyte hemorrhage was observed in rabbits infected with the TS strain (Figure 4G), whereas hepatocyte necrosis was also observed in rabbits infected with the MY strain at 1 dpi (Figure 4H). At 3 dpi, vacuolar degeneration, necrosis, and hemorrhage were significant in the livers of rabbits in both experimental groups (Figure 4I and 4J). At 1 dpi, the spleens of rabbits infected with TS strain exhibited massive lymphocyte loss with diffuse hemorrhaging (Figure 4L), but the reduction of lymphocytes with hemorrhage had decreased significantly by 3 dpi (Figure 4N). Similarly, infected with the MY strain, rabbits showed massive lymphocyte loss with severe hemorrhage at 1 dpi (Figure 4M), which had decreased by 3 dpi (Figure 4O). Intestinal villus rupture was observed in rabbits infected with the TS strain (Figure 4Q), whereas goblet cell enlargement and muco-enteritis were salient in rabbits infected with the MY strain at 1 dpi (Figure 4R). At 3 dpi, intestinal villus shedding and structural destruction had occurred (Figure 4S and 4T). By total contrast, the control group showed no obvious pathological change whatsoever (Figure 4A, 4F, 4K, and 4P).

### Expression of innate immune-related genes in the spleens of infected rabbits

**Figure 5:**
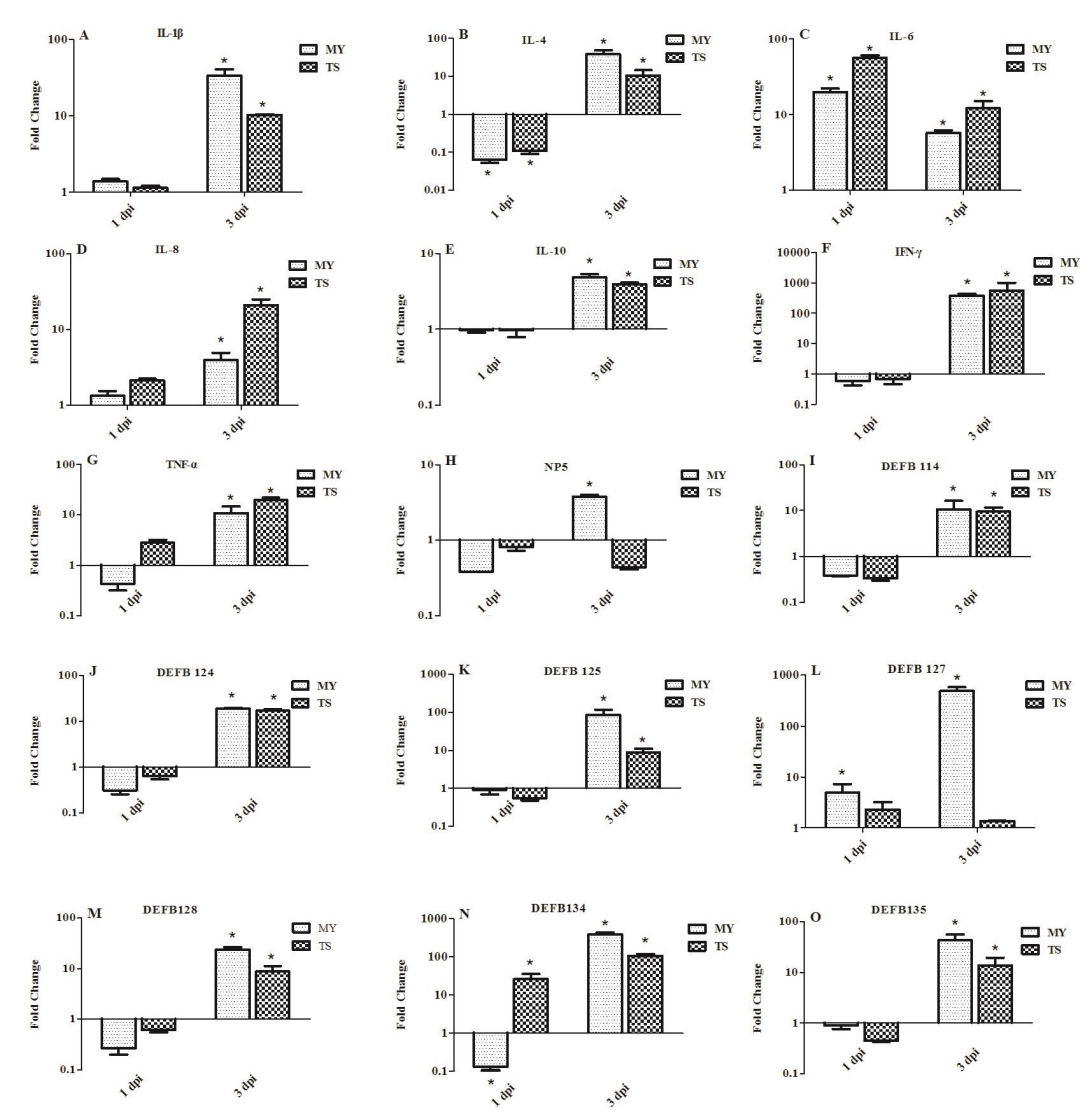
Expression profiles of immune-related genes in the spleen of rabbits at 1 and 3 d postinfection Image **A.** shows IL-1β, **B.** IL-4, **C.** IL-6, **D.** IL-8, **E.** IL-10, **F.** IFN-γ, **G.** TNF-α, **H.** NP5, **I.** DEFB114, **J.** DEFB124, **K.** DEFB125, **L.** DEFB127, **M.** DEFB128, **N.** DEFB134, and **O.** DEFB135. Fold change was calculated from the gene expression of two groups of infected rabbits compared with that of control group. Bars represented the means ± SDs of three independent experiments (five rabbits per experiment); significant differences are indicated with an *.

To investigate the induction of innate immune response in rabbits infected with *E. coli*, the expression of genes involved in such response was examined in the spleen at 1 and 3 dpi. Expressions of pro-inflammatory cytokines IL-1β and IL-8 displayed no significant difference at 1 dpi, yet had significantly upregulated by 3 dpi. For example, at 3 dpi, the expression of IL-1β was upregulated in rabbits infected with the MY and TS strains by 32.21- and 10.22-fold, respectively (*p* < 0.05, Figure 5A), whereas IL-8 was upregulated by 3.94- and 21.07-fold in each respective experimental group (*p* < 0.05, Figure 5D). At 1 dpi, the expression of IL-6 in rabbits infected with the MY and TS strains was also significantly upregulated by 20.06- and 56.80-fold, respectively (*p* < 0.05), yet decreased at 3 dpi by 5.72- and 12.24-fold, also respectively (*p* < 0.05, Figure 5C). By contrast, anti-inflammatory cytokines IL-4 and IL-10 were downregulated at 1 dpi, but upregulated at 3 dpi by 38.86-fold (*p <* 0.05) and 4.86-fold (*p <* 0.05) in rabbits infected with the MY and TS strains, respectively, to a greater extent in the former group than in the latter (Figure 5B and 5E). The expression of IFN-γ was significantly upregulated by 381.83- and 560.38-fold (*p* < 0.05, Figure 5F) in rabbits infected with the MY and TS strains, respectively, whereas at 3 dpi TNF-α showed increases of only 10.81- and 19.62-fold (*p* < 0.05, Figure 5G) in each respective group.

At 1 dpi, the expression of α-defensin (i.e., NP5) and β-defensin (i.e., DEFB114, DEFB124, DEFB125, DEFB127, DEFB128, DEFB134, and DEFB135) displayed no significant changes in rabbits infected with either the MY or TS strain. However, at 3 dpi, those genes showed greater expression in rabbits infected with the MY strain than those in the control group and those infected with the TS strain. At 3 dpi, NP5, DEFB 114, DEFB124, and DEFB128 showed a relatively low expression (Figure 5H, 5I, 5J, and 5M), whereas DEF125, DEFB127, DEFB134, and DEFB135 displayed a change of 3.26-23.62-fold (Figure 5K, 5L, 5N, and 5O). At 3 dpi, the expression of DEFB127 and DEFB134 was especially upregulated, by 491.24- and 382.11-fold, in rabbits infected with the MY strain (*p* < 0.05; Figure 5L and 5O).

### Expression of innate immune-related genes in the livers of infected rabbits

**Figure 6:**
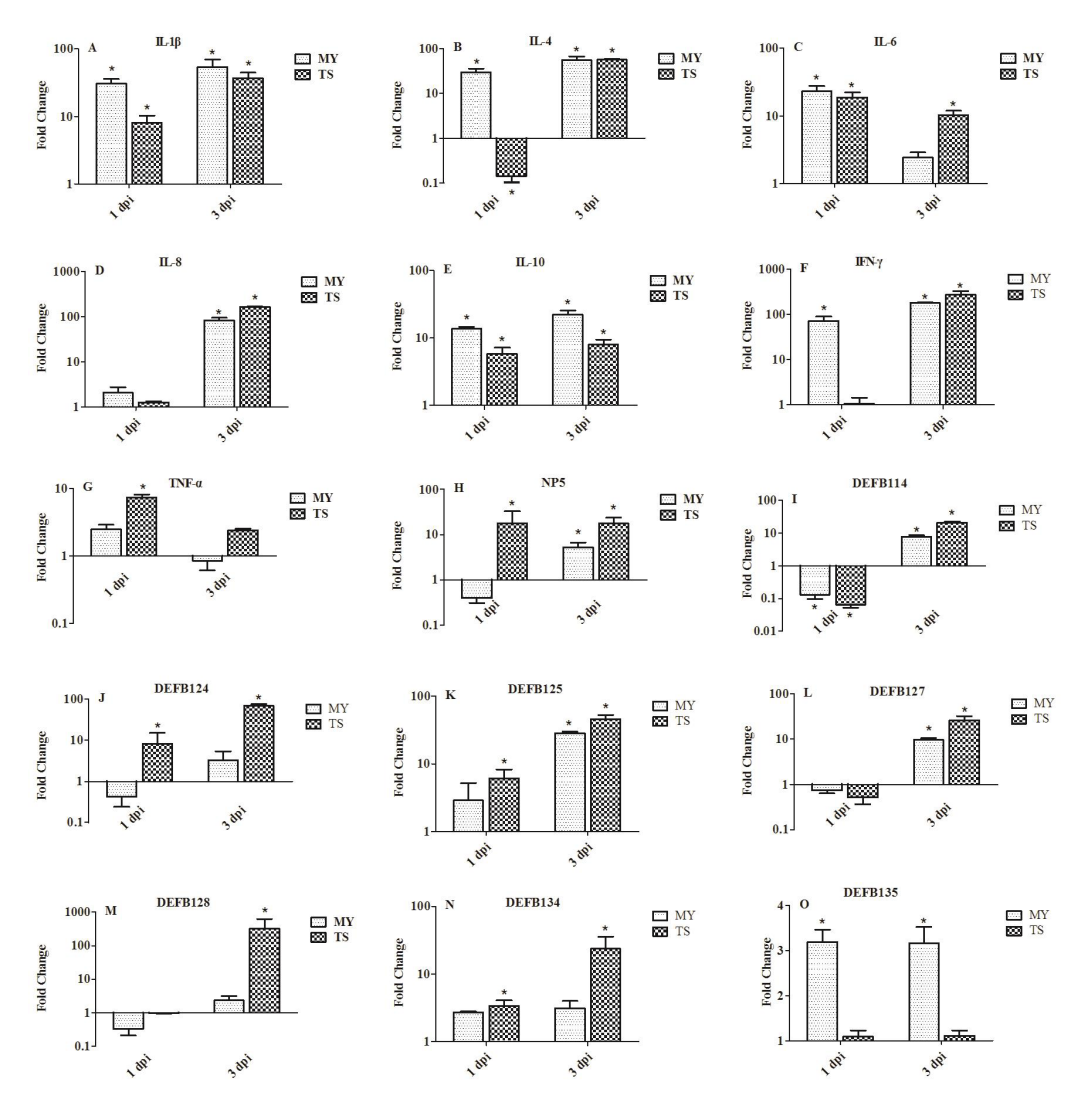
Expression profiles of immune-related genes in the liver of rabbits at 1 and 3 d postinfection Image **A.** shows IL-1β, **B.** IL-4, **C.** IL-6, **D.** IL-8, **E.** IL-10, **F.** IFN-γ, **G.** TNF-α, **H.** NP5, **I.** DEFB114, **J.** DEFB124, **K.** DEFB125, **L.** DEFB127, **M.** DEFB128, **N.** DEFB134, and **O.** DEFB135. Fold change was calculated from the gene expression of two groups of infected rabbits compared with that of control group. Bars represented the means ± SDs of three independent experiments (five rabbits per experiment); significant differences are indicated with an *.

In the livers of rabbits in both groups at 1 dpi, the expression of IL-1β and IL-6 showed elevated expression (Figure 6A and 6C). At 3 dpi, the expression of IL-1β was significantly upregulated, by 54.29- and 37.20-fold, in rabbits infected with the MY and TS strains, respectively (Figure 6A). The expressions of IL-4 and IL-10 in rabbits infected with the MY strain were greater than in rabbits infected with the TS strain (Figure 6B and 6E); the expression of IL-4 was particularly upregulated, by 29.69- and 55.11-fold, in rabbits infected with the MY strain at 1 and 3 dpi, respectively (*p* < 0.05, Figure 6B). IL-8 showed slight variation at 1 dpi, but was significantly upregulated, by 83.12- and 165.82-fold, in rabbits infected with the MY and TS strains, respectively, at 3 dpi (*p* < 0.05, Figure 6D). Furthermore, at 3 dpi, the expression of IFN-γ was upregulated by 179.43- and 272.5-fold in rabbits infected with the MY and TS strains, respectively (*p* < 0.05, Figure 6F). However, at 1 dpi, the expression of TNF-α was upregulated by only 7.34-fold (*p* < 0.05) in rabbits infected with the TS strain, and its expression showed no significant changes in that group of rabbits at 3 dpi (Figure 6G).

Both α- and β-defensin showed greater expressions in rabbits infected with the TS strain than the MY strain, except regarding DEFB135 (Figure 6O). At 1 and 3 dpi, the expression of NP5 was significantly upregulated, by 17.89- and 17.52-fold, respectively, in rabbits infected with the TS strain (*p* < 0.05, Figure 6H). The expression of DEFB114, DEFB127, and DEFB128 were downregulated at 1 dpi and upregulated at 3 dpi (Figure 6I, 6L, 6M). However, at 3 dpi, the expression of DEFB128 was significantly upregulated, by 321.28-fold (*p* < 0.05), in rabbits infected with the TS strain and to a far greater extent than that of other defensins (Figure 6M). At 3 dpi, the expression of DEFB124, DEFB125, and DEFB134 reached 68.16-, 45.61-, and 23.57-fold in rabbits infected with the TS strain (*p* < 0.05, Figure 6J, 6K, 6N).

## DISCUSSION

Increasingly used as a food source, as experimental animals, and as household pets, rabbits nevertheless suffer from diarrhea and even rapid death due to EHEC infection during breeding. In response, clinical symptoms, histopathological aspects, *E. coli* content in tissues, and host innate immune in rabbits were systematically examined in the present study.

MY and TS strains carry different toxin genes, MY strain carries Stx2 and TS strain does not. Therefore, MY and TS strains might have different ability to produce toxin. The survival rate of rabbits infected with the MY strain was lower than those infected with the TS strain. The clinical pathology of diarrhea and sudden death of rabbits was consistent with findings from previous research [31]. Pathological changes in rabbits infected with the MY strain were more serious than those infected with the TS strain at 1dpi and 3dpi. Although the pathogenicity of the MY strain was stronger than that of the TS, *E. coli* content in tissues infected with the TS strain was greater than those infected with the MY strain, except for the spleen. The possible reason is that MY strain grew at a faster rate than the TS strain during the logarithmic growth phase *in vitro*. MY strain can damage the tissues and organs of the host more quickly. The amount of *E. coli* had decreased by 3 dpi, and the decreased level was higher in rabbits infected with the MY strain. This could explain why rabbits infected with the MY strain no longer died after 3 dpi. Bacteria in the heart, liver, spleen, and cecal contents of both *E. coli*-infected rabbits were capable of reaching 10^8^ CFU/g and thus causing extensive clinical symptoms and histopathological lesions, thereby indicating that EHEC had broad tissue tropism, especially in the spleen and liver. Moreover, the *E. coli* content in spleen which is the main immune organ infected with the MY strain was greater than that infected with the TS strain. We hypothesized that the MY strain would cause stronger immunosuppression.

Researches have suggested that the innate immune system is fixed in the genome, and can rapidly mobilize and identify pathogenic microorganisms that induce conserved molecular patterns [32, 33]. When stimulated with pathogenic microorganisms, innate immune responses are implemented and ready to activate natural antibodies and the alternative complement system. Innate immunity relates closely to antimicrobial effector functions provided by local inflammation [34]. In response, the expression of immune-related genes was explored in the spleen and liver of rabbits infected with EHEC in the present study.

Pro-inflammatory cytokines IL-1β and IL-6 cause inflammatory events in order to eliminate pathogenic microorganisms. Previous research has indicated that Stxs can induce the production of IL-8 in HCT-8 cells, since Stxs-mediated ERK1/2 activation is necessary for Stx1-mediated IL-8 expression and can promote the systemic absorption of Stxs, thereby promoting systemic disease [35, 36]. Results of the present study revealed the significant upregulation of IL-8 expression, by 83.12- and 165.82-fold, in the liver during infection with the MY and TS strains, respectively, possibly due to the stimulation of Stxs. Produced following lipopolysaccharide stimulation, TNF-α induces IL-1β, IL-6, and IL-8 secretion. IL-1β at once enhances the expression of IL-6, IL-8, and TNF-α [37]. The results of the present study showed that IL-1β, IL-6, and TNF-α were all significantly induced by MY and TS strains in the spleen at 3 dpi, thereby indicating the activation of the host innate immune response. IL-1β, IL-6, IL-8, and TNF-α promote with each other to form a network of interactions that maintain an appropriate immune response and prevent excessive host immune defense.

In various defense mechanisms, antimicrobial peptides are important evolutionarily conserved components of innate immunity that exist widely in nearly all species, including bacteria, fungi, insects, tunicates, amphibians, crustaceans, birds, fish, and mammals, including humans. Roughly three decades ago, antimicrobial peptides were initially isolated from the lymph of insects, skin of frogs, and granules of mammalian neutrophils and demonstrated the ability to kill bacteria *in vitro* [38]. Our results showed that the α- and β-defensin showed no obvious change induced by MY and TS strains at 1 dpi. It is possible that some pathogenic factors of bacteria may have prevented the expression and activation of defensins. However, the expressions of α- and β-defensin were significantly upregulated, and bacteria in tissues and deaths of rabbits had both declined by 3 dpi. Moreover, the pathological changes in spleen were alleviated at 3 dpi. That phenomenon is consistent with the antibacterial ability of defensins, which inhibit the further reproduction of bacteria in the host. Notably, the expression of α- and β-defensin induced by the MY strain was greater than that induced by the TS strain in the spleen. However, the expression of α- and β-defensin induced by the TS strain was greater than that induced by the MY strain in the liver. Such results could stem from the circumstance that *E. coli* content in spleens infected by the MY strain exceeded that of spleens infected with the TS strain and that *E. coli* content in livers infected with the TS strain was greater than in those infected by the MY strain. In short, more *E. coli* induced the production of defensins.

Altogether, the findings show that pathogenicity of the MY strain was greater than that of the TS strain in rabbits. Two *E. coli* could replicate efficiently in many tissues of rabbits, and pro-inflammatory cytokines IL-1β, IL-6, IL-8, IFN-γ, and TNF-α were all induced in the spleen and liver by the MY and TS strains. The expressions of α- and β-defensin induced by the MY and TS strains differed in the spleen and liver. As such, the present study has pioneered the systematic exploration of the expression of immune-related genes in EHEC-infected rabbits. Its findings also provide useful information about the relationship between EHEC pathogenicity and host immune responses in rabbits.

## MATERIALS AND METHODS

### Ethics statement

This study was carried out in accordance with the recommendations of Shandong Agricultural University Animal Care and Use Committee (no. SDAUA-2015-005).

### Bacterial strains

The EHEC TS and MY strains were isolated from intestine of clinically infected rabbits that developed acute diarrhea. Using eosin-methylene blue medium as the selective medium, and suspected colonies were subjected to staining microscopy and biochemical tests. Then bacterial strains were grown in nutrient broth medium at 37°C. Overnight cultures of bacteria were harvested for DNA extraction using the TIANamp Bacteria DNA Kit (Transgen Biotech Co., Ltd., Beijing, China) according to the manufacturer’s instructions. Following those tests, 16S rDNA sequencing was performed with universal primers 27F (5’-AGAGTTTGATCCTGGCTCAG-3’) and 1492R (5’-GGTTACCTTGTTACGACTT-3’), and the sequences were blasted in NCBI. To further amplify the classical virulence genes of EHEC, the primer pairs of eae, Stx1B, Stx2A and variants, as well as hlyA genes [1] are listed in Table 1.

**Table 1:** Primers used in this study.

Primer name	Sequence(5’-3’)	Purpose
eae F	tgcggcacaacaggcggcga	Gene cloning
eae R	cggtcgccgcaccaggattc	
Stx1B F	cccggatccatgaaaaaaacattattaatagc	Gene cloning
Stx1B R	cccgaattcagctattctgagtcaacg	
Stx2A and variants F	atcctattcccgggagtttacg	Gene cloning
Stx2A and variants R	gcgtcatcgtatacacaggagc	
hlyA F	ggtgcagcagaaaaagttgtag	Gene cloning
hlyA R	tctcgcctgatagtgtttggta	
IL-1β F	tggcacgtatgagctgaaag	RT-PCR
IL-1β R	ggccacaggtatcttgtcgt	
IL-4 F	cactccggcagttctacctc	RT-PCR
IL-4 R	gcagaggttcctgtcgagtc	
IL-6 F	ctgaagacgaccacgatcca	RT-PCR
IL-6 R	aaggacacccgcactccat	
IL-8 F	ctctcttggcaaccttcctg	RT-PCR
IL-8 R	ttgcacagtgaggtccactc	
IL-10 F	aaaagctaaaagccccagga	RT-PCR
IL-10 R	cgggagctgaggtatcagag	
IFN-γ F	ctcgaatttcggtggatgat	RT-PCR
IFN-γ R	agcgtctgactcctttttcg	
TNF-α F	cacttcagggtgatcggc	RT-PCR
TNF-α R	tgcgggtttgctactacg	
DEFB114 F	taccagccacatgctctttg	RT-PCR
DEFB114 R	cctgtcgacacagcaaatct	
DEFB124 F	gcaccaagcaagagtccttc	RT-PCR
DEFB124 R	acgccagagccagctactta	
DEFB125 F	cgtgctgcatctccttaaca	RT-PCR
DEFB125 R	gcgaagcagaaaattgatcc	
DEFB127 F	cccacagtaaccgagcaact	RT-PCR
DEFB127 R	gctgaggcagcagtatctcc	
DEFB128 F	gggctcaaggctttctcttt	RT-PCR
DEFB128 R	aaatctcgcctagcttgcac	
DEFB134 F	agcctgtctgcctggagtag	RT-PCR
DEFB134 R	gatgaggagaggcttcatgg	
DEFB135 F	gctgcatctccaaatccaat	RT-PCR
DEFB155 R	tagtgggatggtgcaactga	
NP 5 F	aggcaggcgtgttctgtact	RT-PCR
NP 5 R	ggtctccacgcaaataagga	
GAPDH F	aggtcatccacgaccacttc	RT-PCR
GAPDH R	gtgagtttcccgttcagctc	

**Table 2:** Amplification of virulence factors.

Strain	Eae	Stx1	Stx2	HlyA
TS	+	+	-	+
MY	+	+	+	+

### Growth curve

Overnight cultures of TS and MY strains in amounts of 1 mL (10^9^ CFU) were introduced into 100 mL nutrient broth medium. The new liquid medium were incubated at 37°C for 24 h, and calculations of *E. coli* content every 2 h were used to develop a growth curve.

### Animal experiments

A group of 120 healthy weaned rabbits 35 d old were randomly separated into three treatment groups (*n* = 40) and given sufficient feed and water. Challenge tests were conducted by using intraperitoneal injections of 1.0 mL of each *E. coli* bacterial suspension (10^8^ CFU/mL). Rabbits in the control group were inoculated with 1.0 mL of phosphate-buffered saline. From each group, 10 rabbits were randomly chosen to provide data used to calculate the survival rate of each group. The survival rate of the rabbits was calculated at the end of the experiment. At 1 and 3 dpi, five rabbits in each group were euthanized, the hearts, livers, spleens, brains and colons were collected, and part of the tissues were fixed with 4% paraformaldehyde solution to study histopathological changes, whereas the others parts were used for RNA extraction. The fresh and sterile heart, liver, spleen, brain, and cecal content were collected to gauge *E. coli* content as the following: tissue samples were weighed, suspended in PBS (1ml/g). Serial 10-fold dilutions were plated onto nutrient agar to calculate CFU per gram in each organ. All rabbits were handled according to appropriate biosecurity guidelines. All experiments were performed in a biosafety level-2 laboratory.

### Quantitative real-time PCR

Total RNA was extracted from the spleen and liver of rabbits using the RNeasy plus Mini Kit (Qiagen, Hilden, Germany) according to the manufacturer’s instructions. For quantitative real-time (qRT) PCR, total RNA (1 µg) was reverse transcribed with *TransScript*^R^ One-step gDNA Removal and cDNA Synthesis SuperMix (Transgen Biotech Co., Ltd., Beijing, China). The synthesized cDNA was stored at -20°C until further use. qRT-PCR was performed using *TransStart*^R^ Tip Green qPCR SuperMix (+Dye II) (Transgen Biotech Co., Ltd., Beijing, China). Primers of IL-6 and TNF-α used for qRT-PCR have previously been reported [39]. Other primers used for qRT-PCR were designed with Primer 3 software (http://bioinfo.ut.ee/primer3-0.4.0/) based on target sequences previously reported (Table 1). qRT-PCR was performed using the 7500 Fast Real-Time PCR System (Applied Biosystems, Carlsbad, CA, USA). PCR involved a cycle at 94°C for 30 s, followed by 40 cycles at 94°C for 5 s and 60°C for 34 s. Dissociation curves of the products were ultimately developed.

### Statistical analysis

The relative expressions of genes were calculated using the 2^−ΔΔCt^ method. The housekeeping gene GAPDH was used as an endogenous control to normalize the expression of target genes. Data were analyzed with the non-parametric Mann-Whitney U test, and the survival rate of rabbits was analyzed using the Kaplan-Meier method. All statistical analyzes were performed with GraphPad Prism 5.0 (GraphPad Software Inc., San Diego, CA, USA), with statistical significance set at *p* < 0.05.
